# RNA sequencing data for heat stress response in isolated *medicago truncatula* seed tissues

**DOI:** 10.1016/j.dib.2021.106726

**Published:** 2021-01-21

**Authors:** Zhijuan Chen, Benoit Ly Vu, Olivier Leprince, Jerome Verdier

**Affiliations:** Institut de Recherche en Horticulture et Semences-UMR1345, Université d'Angers, INRAE, Institut Agro, SFR 4207 QuaSaV, 49071, Beaucouzé, France

**Keywords:** RNA sequencing, Seed maturation, Heat stress, Seed quality, Embryo, Endosperm, Seed coat

## Abstract

Legumes are important crop species as they produce highly nutritious seeds for human food and animal feed. In grain legumes, sub-optimal conditions affect seed developmental timing leading to impairment of seed quality traits acquired during seed maturation. To understand the molecular mechanisms of heat stress response in legume seeds, we analysed transcriptome changes of three seed tissues (i.e. em bryo, endosperm and seed coat) at four developmental stages, during seed maturation, from seed filling to mature dry seeds, collected under optimal and heat stress conditions in the model legume, *Medicago truncatula* (reference genotype A17). The total RNA sequencing generated a dataset of 48 samples, representing more than 57 Gb fastq raw data. Mapping, quantification and annotation of the data were based on fifth release of *Medicago truncatula* genome and provided expression profiles of 44,473 transcripts in seed tissues at different developmental stages and under optimal and stress conditions. Time-course and pairwise comparisons between optimal and stress conditions showed that 9182, 8315 and 3481 genes were differentially expressed due to heat stress in embryo, endosperm and seed coat respectively. Moreover, it highlighted a common set of 975 genes that were differentially expressed in all the seed tissues.

## Specifications Table

**Table:** 

Subject	Agricultural and Biological Sciences
Specific subject area	Omics: Transcriptomics Plant Science: Plant Physiology
Type of data	Tables Figures
How data were acquired	Total RNA samples were sent to BGI, Hong Kong, for library preparation. Libraries were constructed following a custom protocol from samples that passed quality controls (mass > 2 µg, concentration>80 ng/microl, OD260/280 ≅ 2.00, OD260/230 ≅ 2.20, RIN>6.5, 28S/18S<1.0, baseline smooth). After mRNA enrichment by rRNA depletion and oligo dT selection, RNA was fragmented and reverse transcribed to double-strand cDNA (dscDNA) by N6 random primer. The synthesized cDNA was subjected to end-repair and then was 3′ adenylated. Adaptors were ligated to the ends of these 3′ adenylated cDNA fragments. The ligation products were purified and many rounds of PCR amplification were performed to enrich the purified cDNA template using PCR primer, splint oligo and DNA ligase, followed by sequencing on BGISEQ-500 platform, generating an average 24 M reads of 50 bp per sample.
Data format	Filtered raw reads (FASTQ) Analysed RNA-seq data files (counts and DEG lists)
Parameters for data collection	Total RNAs were extracted from isolated embryo, endosperm and seed coat of four developmental stages of *Medicago truncatula* (A17) seeds that were harvested during maturation phase before and after acquisition of desiccation tolerance (respectively S1 and S2) and at the onset and after longevity acquisition (respectively S3 and S4) under standard temperature (20 °C day/ 18 °C night) and under high temperature (26 °C/24 °C).
Description of data collection	RNA-seq dataset was collected from single-end sequencing of cDNA libraries using BGISeq500 platform with 50 bp reads. Raw reads were filtered to remove adapters and low-quality reads, then mapped to *Medicago truncatula* reference transcriptome (version 5). Total mapped reads and number of transcripts (counts and TPM) were estimated using Salmon algorithm. Differential expressions of genes between standard and heat stress conditions were calculated using ImpulseDE2 and DEseq2 algorithms.
Data source location	Institution: Growth chambers from the Institut de Recherche en Horticulture et Semences, INRAE City: Beaucouzé Country: France
Data accessibility	Public Repository: Repository name: NCBI GEO Data identification number: GSE160725 Direct URL to data: https://www.ncbi.nlm.nih.gov/geo/query/acc.cgi?acc=GSE160725

## Value of the Data

• These data represent valuable seed transcriptome dataset of heat stress in the model legume *Medicago truncatula* because it has been generated from isolated seed tissues (embryo, endosperm and seed coat) along the whole seed maturation.
• These data are useful resources for scientific communities working on seed and legume seed quality but also on plant stress biology to understand specific and common stress response pathways.
• These data provide new insights about molecular processes affected during heat stress in different seed tissues and candidate genes and molecular markers to predict seed quality in sub-optimal conditions.

## 1 Data Description

**Fig. 1:**
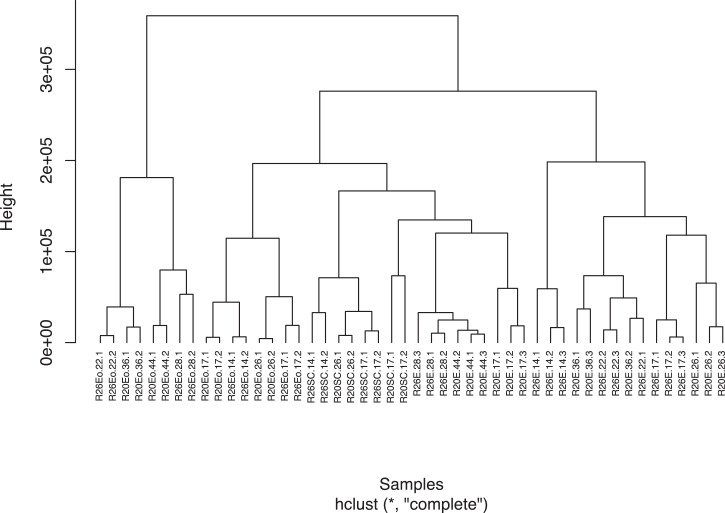
Hierarchical cluster analysis based on dissimilarity scores obtained from the TPM values of the 48 samples to validate reproducibility of replicates.

**Fig. 2:**
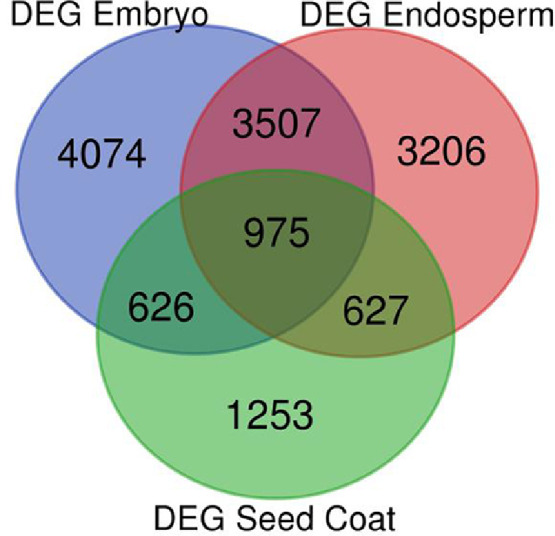
Venn diagram with differentially expressed genes (DEGs, adjusted p-values below 1%) in the three seed tissues between optimal and heat stress conditions.

This manuscript presents a transcriptomic dataset obtained from dissected seed tissues (embryo, endosperm and seed coat) at four developmental stages of *Medicago truncatula* (A17) seeds during maturation phase [i.e. before and after desiccation tolerance (respectively S1 and S2) and at the onset and after longevity acquisition (respectively S3 and S4)] produced under standard temperature (20 °C day/ 18 °C night) and heat stress (26 °C/24 °C). Table 1 displays all sample names with information regarding seed tissues, treatments, developmental stages and numbers of 50 bp reads sequenced per sample. Figure S1 provides sequence quality histograms obtained from FastQC [1] and merged into a single graphic using MultiQC [2]. All 48 samples displayed Phred quality scores about 35, which corresponds to a base calling accuracy of about 99.95%. Salmon algorithm [3] was used to map and quantify raw reads to the reference *Medicago truncatula* transcriptome version 5 and corresponding count table is provided as Table S1. After mapping and quantification, these 48 samples were, then, hierarchically clustered based on their dissimilarity scores to validate reproducibility of replicates (Fig. 1). From this count table, differentially expressed genes were identified using time-course comparisons of the four developmental stages for embryo (Table S2) and endosperm (Table S3); and using pairwise comparisons of the two developmental stages for seed coat (Table S4). Fig. 2 summarizes the differentially expressed genes (DEGs) in the three seed tissues between optimal and heat stress conditions (False Discovery Rate, FDR<1%) and highlights a common set of 975 DEGs within the three seed tissues.

**Table 1:** Summary of sample files with corresponding information related to seed tissues, seed developmental stages, growth conditions and numbers of cleaned 50 bp reads used for RNA-seq mapping.

Tissues	Sample	Numbers of 50 bp reads	Sample	Numbers of 50 bp reads	Stages
Embryo	R20E-17–1	20,452,270	R26E-14–1	20,822,668	S1
Embryo	R20E-17–2	20,465,064	R26E-14–2	20,786,562	S1
Embryo	R20E-17–3	20,364,545	R26E-14–3	20,718,145	S1
Embryo	R20E-26–1	20,452,569	R26E-17–1	20,666,800	S2
Embryo	R20E-26–2	20,137,018	R26E-17–2	20,704,789	S2
Embryo	R20E-26–3	21,967,973	R26E-17–3	20,753,459	S2
Embryo	R20E-36–1	22,003,911	R26E-22–1	20,717,753	S3
Embryo	R20E-36–2	22,156,003	R26E-22–2	20,786,906	S3
Embryo	R20E-36–3	20,183,128	R26E-22–3	20,644,844	S3
Embryo	R20E-44–1	20,760,225	R26E-28–1	20,649,142	S4
Embryo	R20E-44–2	20,897,210	R26E-28–2	20,749,797	S4
Embryo	R20E-44–3	20,744,207	R26E-28–3	20,778,364	S4
Embryo	R20Eo-17–1	20,677,185	R26Eo-14–1	20,303,572	S1
Endosperm	R20Eo-17–2	20,659,667	R26Eo-14–2	20,257,351	S1
Endosperm	R20Eo-26–1	20,815,605	R26Eo-17–1	20,342,065	S2
Endosperm	R20Eo-26–2	20,174,987	R26Eo-17–2	20,221,552	S2
Endosperm	R20Eo-36–1	20,332,236	R26Eo-22–1	20,288,004	S3
Endosperm	R20Eo-36–2	20,129,499	R26Eo-22–2	20,463,956	S3
Endosperm	R20Eo-44–1	20,829,827	R26Eo-28–1	20,371,220	S4
Endosperm	R20Eo-44–2	20,810,635	R26Eo-28–2	20,405,449	S4
Seed Coat	R20SC-17–1	40,072,723	R26SC-14–1	41,944,942	S1
Seed Coat	R20SC-17–2	56,846,997	R26SC-14–2	42,596,392	S1
Seed Coat	R20SC-26–1	39,483,027	R26SC-17–1	22,920,874	S2
Seed Coat	R20SC-26–2	48,482,527	R26SC-17–2	34,721,666	S2
	CONTROL CONDITION		STRESS CONDITION

## 2 Experimental Design, Materials and Methods

### 2.1 Plant growth conditions and seed tissue sampling

Medicago plants were grown under standard conditions (20 °C/18 °C, 16 h photoperiod) in growth chamber. At flowering time, half of plants were kept at same optimal conditions (20 °C/18 °C, 16 h photoperiod) and half were grown under heat stress condition (26 °C/24 °C, 16 h photoperiod). Developing and mature seeds were collected from standard and stress conditions, seed tissues were quickly dissected then immediately frozen in liquid nitrogen before RNA extraction. According to our previous study [4], four seed developmental stages were collected under standard conditions at 17 days after pollination (DAP), 26 DAP, 36DAP and 44 DAP and under heat stress conditions at corresponding developmental stages at 14 DAP, 17 DAP, 22 DAP and 28 DAP. These four developmental stages were shown to be the beginning of seed maturation and before acquisition of desiccation tolerance (S1), after the acquisition of desiccation tolerance and during seed filling (S2), at the onset of the acquisition of longevity and at the end of seed filling (S3) and mature seed (S4) according to our previous study [4].

### 2.2 RNA isolation and sequencing

Total RNAs were extracted from embryo and endosperm that were collected at four stages during seed maturation under standard and heat stress conditions. RNAs were only extracted from S1 and S2 for seed coat, as during late maturation stage seed coat is dying and does not provide good quality RNA. All RNA extractions were performed using MACHEREY-NAGEL NucleoSpin® RNA Plus kit. RNA quality was checked using a nanodrop spectrophotometer ND-1000 (NanoDrop Technologies) and a 2100 Bioanalyzer (Agilent Technologies, Santa Clara, CA, USA). All samples with good qualities (260/280 and 260/230 absorbance ratio >2; RNA Integrity Number, RIN>8; 28S/18S>1.7) were sent to Beijing Genomics Institute (https://www.bgi.com) (Hong Kong) for library preparation and sequencing on BGISEQ-500 platform, generating an average of 20 M reads of 50 bp per sample.

### 2.3 RNA-seq data analyses

After quality control of fastq files using FastQC [1], high-quality reads were mapped onto the reference Medicago A17 transcriptome version 5 (https://medicago.toulouse.inra.fr/MtrunA17r5.0-ANR/downloads/1.7/MtrunA17r5.0-ANR-EGN-r1.7.fastaFiles.zip) (Table S5) [5] and transcript abundances were quantified with Salmon algorithm (version 0.14.1) [3] using the quasi-mapping mode and the ‘–validateMappings’, ‘–useVBOpt’ and ‘–seqBias’ options. Reproducibility of replicates was validated by a hierarchical cluster analysis based on dissimilarity scores obtained from the normalized raw count values of the 48 samples using the ‘cpm’, ‘dis’ and ‘hclust’ functions in R. Differentially expressed genes (DEGs) were identified by time-course comparisons of the four developmental stages of embryo and endosperm using ImpulseDE2 [6] and by pair-wise comparisons of the two developmental stages of seed coat using DESeq2 [7]. All transcripts with change in expression showing adjusted p-values below 1% (no fold change cutoff) were considered as differentially expressed between standard and heat stress conditions and displayed on a Venn Diagram performed from the website: http://bioinformatics.psb.ugent.be/webtools/Venn/.

## Ethics Statement

This work does not contain any studies with human or animal subjects.

## CRediT Author Statement

**Zhijuan Chen:** Investigation, Methodology, Visualization, Writing - Original draft preparation; **Benoit Ly Vu:** Methodology; **Olivier Leprince:** Conceptualization, Reviewing and Editing; **Jerome Verdier:** Supervision, Conceptualization, Data curation, Writing - Reviewing and Editing.

## Declaration of Competing Interest

The authors declare that they have no known competing financial interests or personal relationships which have or could be perceived to have influenced the work reported in this article.

## Appendix Supplementary materials

Table S1: Count table of mapped reads from the 48 samples using Salmon algorithm. Image, application 1

Table S2: List of differentially expressed genes in Embryo using time-course comparisons of the four developmental stages obtained from ImpulseDE2 algorithm. Image, application 2

Table S3: List of differentially expressed genes in Endosperm using time-course comparisons of the four developmental stages obtained from ImpulseDE2 algorithm. Image, application 3

Table S4: List of differentially expressed genes in Seed Coat using pairwise comparisons of the two developmental stages obtained from DEseq2 algorithm. Image, application 4

Table S5: Reference transcriptome, from *Medicago truncatula* genome version 5, used in this study and available at https://medicago.toulouse.inra.fr/MtrunA17r5.0-ANR/downloads/1.7/MtrunA17r5.0-ANR-EGN-r1.7.fastaFiles.zip Image, application 5

Figure S1: Overview of the Phred quality values across all bases at each position in the fastq files from the 48 samples obtained from FastQC and MultiQC analyses. Image, application 6

Image, application 7

Image, application 8

Image, application 9

Image, application 10

Image, application 11

Image, application 12

Image, application 13

Image, application 14

Image, application 15

Image, application 16

## Footnotes

Supplementary material associated with this article can be found in the online version at doi:10.1016/j.dib.2021.106726.
