# Supplementary material for: RNA sequencing data for heat stress response in isolated medicago truncatula seed tissues
Source: Data Brief. 2021 Jan 21;35:106726. doi: 10.1016/j.dib.2021.106726 (PMC7856423; doi:10.1016/j.dib.2021.106726)
Supplement: Supplementary file 6 — Figure S1: Overview of the Phred quality values across all bases at each position in the fastq files from the 48 samples obtained from FastQC and MultiQC analyses. [file mmc6.zip › mmc6.html]

MultiQC Report


# Toggle navigation v1.0.dev0

- General Stats
- FastQC
  - Sequence Quality Histograms
  - Per Sequence Quality Scores
  - Per Base Sequence Content
  - Per Sequence GC Content
  - Per Base N Content
  - Sequence Length Distribution
  - Sequence Duplication Levels
  - Overrepresented sequences
  - Adapter Content

Toolbox

### MultiQC Toolbox

#### Apply Highlight Samples

+

Regex mode off
help
 Clear

#### Apply Rename Samples

+

Click here for bulk input.

Paste two columns of a tab-delimited table here (eg. from Excel).

First column should be the old name, second column the new name.

Add

Regex mode off
help
 Clear

#### Apply Show / Hide Samples

Hide matching samples

Show only matching samples

+

Regex mode off
help
 Clear

#### Export Plots

- Images
- Data

px

px

Aspect ratio

PNG
JPEG
SVG

Plot scaling

X

Download the raw data used to create the plots in this report below:

Format:

Tab-separated
Comma-separated
JSON

Note that additional data was saved in `multiqc_data` when this report was generated.

---

##### Choose Plots

 All
 None

---


   Download Plot Images

If you use plots from MultiQC in a publication or presentation, please cite:

> **MultiQC: Summarize analysis results for multiple tools and samples in a single report**  
> *Philip Ewels, Måns Magnusson, Sverker Lundin and Max Käller*  
> Bioinformatics (2016)  
> doi: 10.1093/bioinformatics/btw354  
> PMID: 27312411

#### Save Settings

You can save the toolbox settings for this report to the browser.

 Save


---

#### Load Settings

Choose a saved report profile from the dropdown box below:

[ select ]

Load
 Delete

#### About MultiQC

This report was generated using MultiQC, version 1.0.dev0

You can see a YouTube video describing how to use MultiQC reports here:
https://youtu.be/qPbIlO\_KWN0

For more information about MultiQC, including other videos and
extensive documentation, please visit http://multiqc.info

You can report bugs, suggest improvements and find the source code for MultiQC on GitHub:
https://github.com/ewels/MultiQC

MultiQC is published in Bioinformatics:

> **MultiQC: Summarize analysis results for multiple tools and samples in a single report**  
> *Philip Ewels, Måns Magnusson, Sverker Lundin and Max Käller*  
> Bioinformatics (2016)  
> doi: 10.1093/bioinformatics/btw354  
> PMID: 27312411

# 

A modular tool to aggregate results from bioinformatics analyses across many samples into a single report.

Report generated on 2020-11-03, 15:11 based on data in:
`/media/seedver/BASIS/RNA-seq/all_fastq/fastqc`

---

×
don't show again

**Welcome!** Not sure where to start?  
Watch a tutorial video
  *(6:06)*

## General Statistics

 Copy table

 Sort by highlight
Showing 48/48 rows and 3/5 columns.

| Sample Name | % Dups | % GC | Length | % Failed | M Seqs |
| --- | --- | --- | --- | --- | --- |
| R20E-17-1 | 67.0% | 44% | 50 | 20% | 20.5 |
| R20E-17-2 | 67.0% | 43% | 50 | 20% | 20.5 |
| R20E-17-3 | 67.3% | 43% | 50 | 20% | 20.4 |
| R20E-26-1 | 79.5% | 45% | 50 | 10% | 20.5 |
| R20E-26-2 | 80.5% | 45% | 50 | 10% | 20.1 |
| R20E-26-3 | 79.3% | 45% | 50 | 10% | 22.0 |
| R20E-36-1 | 67.9% | 43% | 50 | 20% | 22.0 |
| R20E-36-2 | 71.9% | 44% | 50 | 10% | 22.2 |
| R20E-36-3 | 68.0% | 43% | 50 | 20% | 20.2 |
| R20E-44-1 | 70.0% | 43% | 50 | 20% | 20.8 |
| R20E-44-2 | 70.0% | 43% | 50 | 20% | 20.9 |
| R20E-44-3 | 70.5% | 43% | 50 | 10% | 20.7 |
| R20Eo-17-1 | 51.5% | 41% | 50 | 20% | 20.7 |
| R20Eo-17-2 | 51.4% | 41% | 50 | 20% | 20.7 |
| R20Eo-26-1 | 54.5% | 41% | 50 | 20% | 20.8 |
| R20Eo-26-2 | 49.8% | 41% | 50 | 10% | 20.2 |
| R20Eo-36-1 | 58.8% | 41% | 50 | 20% | 20.3 |
| R20Eo-36-2 | 51.1% | 41% | 50 | 20% | 20.1 |
| R20Eo44-1 | 60.7% | 41% | 50 | 20% | 20.8 |
| R20Eo44-2 | 59.2% | 41% | 50 | 20% | 20.8 |
| R20SC-17-1 | 58.5% | 41% | 50 | 20% | 40.1 |
| R20SC-17-2 | 66.1% | 41% | 50 | 20% | 56.8 |
| R20SC-26-1 | 61.8% | 41% | 50 | 20% | 39.5 |
| R20SC-26-2 | 67.1% | 41% | 50 | 20% | 48.5 |
| R26E-14-1 | 72.4% | 45% | 50 | 10% | 20.8 |
| R26E-14-2 | 72.1% | 44% | 50 | 10% | 20.8 |
| R26E-14-3 | 76.2% | 44% | 50 | 10% | 20.7 |
| R26E-17-1 | 79.8% | 44% | 50 | 10% | 20.7 |
| R26E-17-2 | 80.1% | 44% | 50 | 10% | 20.7 |
| R26E-17-3 | 79.5% | 44% | 50 | 10% | 20.8 |
| R26E-22-1 | 74.8% | 43% | 50 | 10% | 20.7 |
| R26E-22-2 | 75.8% | 44% | 50 | 10% | 20.8 |
| R26E-22-3 | 76.6% | 44% | 50 | 10% | 20.6 |
| R26E-28-1 | 69.8% | 43% | 50 | 10% | 20.6 |
| R26E-28-2 | 70.3% | 43% | 50 | 10% | 20.7 |
| R26E-28-3 | 63.6% | 43% | 50 | 20% | 20.8 |
| R26Eo-14-1 | 54.3% | 41% | 50 | 20% | 20.3 |
| R26Eo-14-2 | 54.5% | 41% | 50 | 20% | 20.3 |
| R26Eo-17-1 | 58.4% | 41% | 50 | 20% | 20.3 |
| R26Eo-17-2 | 57.5% | 41% | 50 | 20% | 20.2 |
| R26Eo-22-1 | 61.5% | 41% | 50 | 20% | 20.3 |
| R26Eo-22-2 | 60.9% | 42% | 50 | 20% | 20.5 |
| R26Eo-28-1 | 60.9% | 41% | 50 | 20% | 20.4 |
| R26Eo-28-2 | 59.8% | 42% | 50 | 20% | 20.4 |
| R26SC-14-1 | 63.5% | 41% | 50 | 20% | 41.9 |
| R26SC-14-2 | 63.5% | 41% | 50 | 20% | 42.6 |
| R26SC-17-1 | 61.1% | 41% | 50 | 20% | 22.9 |
| R26SC-17-2 | 63.9% | 41% | 50 | 20% | 34.7 |

×

#### General Statistics: Columns

Uncheck the tick box to hide columns. Click and drag the handle on the left to change order.

Show All
Show None

| Sort | Visible | Group | Column | Description | ID | Scale |
| --- | --- | --- | --- | --- | --- | --- |
| || |  | FastQC | % Dups | % Duplicate Reads | `percent_duplicates` | None |
| || |  | FastQC | % GC | Average % GC Content | `percent_gc` | None |
| || |  | FastQC | Length | Average Sequence Length (bp) | `avg_sequence_length` | None |
| || |  | FastQC | % Failed | Percentage of modules failed in FastQC report (includes those not plotted here) | `percent_fails` | None |
| || |  | FastQC | M Seqs | Total Sequences (millions) | `total_sequences` | read\_count |

Close

## FastQC

FastQC is a quality control tool for high throughput sequence data, written by Simon Andrews at the Babraham Institute in Cambridge.

### Sequence Quality Histograms

The mean quality value across each base position in the read. See the FastQC help.

loading..

---

### Per Sequence Quality Scores

The number of reads with average quality scores. Shows if a subset of reads has poor quality. See the FastQC help.

loading..

---

### Per Base Sequence Content

The proportion of each base position for which each of the four normal DNA bases has been called. See the FastQC help.

Click a heatmap row to see a line plot for that dataset.

##### *rollover for sample name*

Position: -

%T: -

%C: -

%A: -

%G: -

---

### Per Sequence GC Content

The average GC content of reads. Normal random library typically have a roughly normal distribution of GC content. See the FastQC help.

Percentages
Counts

loading..

---

### Per Base N Content

The percentage of base calls at each position for which an N was called. See the FastQC help.

loading..

---

### Sequence Length Distribution

All samples have sequences of a single length (50bp).

---

### Sequence Duplication Levels

The relative level of duplication found for every sequence. See the FastQC help.

loading..

---

### Overrepresented sequences

The total amount of overrepresented sequences found in each library. See the FastQC help for further information.

loading..

---

### Adapter Content

The cumulative percentage count of the proportion of your library which has seen each of the adapter sequences at each position. See the FastQC help. Only samples with ≥ 0.1% adapter contamination are shown.

No samples found with any adapter contamination > 0.1%

**MultiQC v1.0.dev0**
- Written by Phil Ewels,
available on GitHub.

This report uses HighCharts,
jQuery,
jQuery UI,
Bootstrap,
chroma.js,
FileSaver.js and
clipboard.js.

×

### Plot Table Data

Select Column

Select Column

Please select two table columns.

Close

×

### Regex Help

Toolbox search strings can behave as regular expressions (regexes). Click a button below to see an example of it in action. Try modifying them yourself in the text box.

`^` (start of string)
`$` (end of string)
`[]` (character choice)
`\d` (shorthand for `[0-9]`)
`\w` (shorthand for `[0-9a-zA-Z_]`)
`.` (any character)
`\.` (literal full stop)
`()` `|` (group / separator)
`*` (prev char 0 or more)
`+` (prev char 1 or more)
`?` (prev char 0 or 1)
`{}` (char num times)
`{,}` (count range)

```
samp_1
samp_1_edited
samp_2
samp_2_edited
samp_3
samp_3_edited
prepended_samp_1
tmp_samp_1_edited
tmpp_samp_1_edited
tmppp_samp_1_edited
#samp_1_edited.tmp
samp_11
samp_11111
```

See regex101.com for a more heavy duty testing suite.

Close
