# Supplementary material for: RNA sequencing data for heat stress response in isolated medicago truncatula seed tissues
Source: Data Brief. 2021 Jan 21;35:106726. doi: 10.1016/j.dib.2021.106726 (PMC7856423; doi:10.1016/j.dib.2021.106726)
Supplement: Supplementary file 8 [file mmc8.zip › mmc8.htm]

**Jerome Verdier**

**Researcher INRAE IRHS**

SEED (Seed, Epigenetics, Environment and Development) Lab

---

INRAE - Research Institute in Horticulture and Seeds (IRHS)

Batiment A - Campus du vegetal

42 rue Georges Morel

49071 Beaucouze, France  
Telephone +33 249 180 473

https://www6.angers-nantes.inra.fr/irhs\_eng/

Follow us: **IRHS Seed Lab**
