# Supplementary figures and images for: RNA sequencing data for heat stress response in isolated medicago truncatula seed tissues
Source: Data Brief. 2021 Jan 21;35:106726. doi: 10.1016/j.dib.2021.106726 (PMC7856423; doi:10.1016/j.dib.2021.106726)

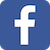

Supplement: Supplementary file 13 [file mmc13.zip › mmc13.png]

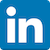

Supplement: Supplementary file 14 [file mmc14.zip › mmc14.png]

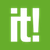

Supplement: Supplementary file 15 [file mmc15.zip › mmc15.png]

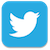

Supplement: Supplementary file 16 [file mmc16.zip › mmc16.png]
