# Supplementary material for: RNA sequencing data for heat stress response in isolated medicago truncatula seed tissues
Source: Data Brief. 2021 Jan 21;35:106726. doi: 10.1016/j.dib.2021.106726 (PMC7856423; doi:10.1016/j.dib.2021.106726)
Supplement: Supplementary file 12 [file mmc12.zip › mmc12.htm]

> On 8 Jan 2021, at 09:58, neha.shandilya@reedelsevier.com wrote:
>
>   
>
> --------------------   
> Our reference: DIB 106726  
> Article reference: DIB\_DIB-D-20-02212  
> Article title:  RNA sequencing data for heat stress response in isolated Medicago truncatula seed tissues  
> To be published in: Data in Brief  
> --------------------   
>   
> Dear Dr Verdier,  
>   
> Congratulations on having your article accepted.   
>   
> We have now received your manuscript in production and would like to begin the typesetting process.  
>   
> Unfortunately we have encountered a problem with the electronic files you provided and cannot process your article further until the following issues are resolved:  
>   
> \*  Table 1 coming in picture format. Also Table S1 is missing from received input files. Kindly provide the samein editable format.  
>   
> We would be grateful if you could kindly address the problem as quickly as possible, ideally within 48 hours, by replying to this message.  
>   
> Further information on acceptable file formats can be found at http://www.elsevier.com/guidepublication.  
>   
> Please quote the reference for your article, DIB 106726, in all of your messages to us.    
>   
> Thank you for your help with this issue; I look forward to hearing from you soon.  
>   
> Kind regards,  
>   
> Neha Shandilya  
> Data Administrator  
> Elsevier  
> E-Mail: neha.shandilya@reedelsevier.com  
>   
> --------------------   
> HAVE QUESTIONS OR NEED ASSISTANCE?   
>   
> For further assistance, please visit our Customer Support site, where you can search for solutions on a range of topics, such as Open Access or payment queries, and find answers to frequently asked questions. You can also talk to our customer support team by phone 24 hours a day from Monday-Friday and 24/7 by live chat and email.   
>   
> Get started here: http://service.elsevier.com/app/home/supporthub/publishing   
> --------------------   
> Copyright © 2015 Elsevier B.V. | Privacy Policy http://www.elsevier.com/privacypolicy   
> Elsevier Limited, The Boulevard, Langford Lane, Kidlington, Oxford, OX5 1GB, United Kingdom, Registration No. 1982084
